# Insulin Delivery to the Brain *via* the Nasal Route: Unraveling the Potential for Alzheimer's Disease Therapy

**DOI:** 10.1007/s13346-024-01558-1

**Published:** 2024-03-05

**Authors:** Chun Yuen Jerry Wong, Alberto Baldelli, Camilla M. Hoyos, Ole Tietz, Hui Xin Ong, Daniela Traini

**Affiliations:** 1 Respiratory Technology, Woolcock Institute of Medical Research, Sydney, NSW 2037 Australia; 2 Faculty of Medicine and Health Sciences, Macquarie Medical School, Macquarie University, Sydney, NSW 2109 Australia; 3 Faculty of Land and Food Systems, The University of British Columbia, 2357 Main Mall, Vancouver, BC V6T 1Z4 Canada; 4 CIRUS Centre for Sleep and Chronobiology, Woolcock Institute of Medical Research, Sydney, NSW 2037 Australia; 5 Dementia Research Centre, Faculty of Medicine and Health Sciences, Macquarie Medical School, Macquarie University, Sydney, NSW 2109 Australia

**Keywords:** Insulin, Blood-brain barrier, Nasal Delivery, Peptides, Alzheimer’s Disease

## Abstract

This comprehensive review delves into the potential of intranasal insulin delivery for managing Alzheimer's Disease (AD) while exploring the connection between AD and diabetes mellitus (DM). Both conditions share features of insulin signalling dysregulation and oxidative stress that accelerate inflammatory response. Given the physiological barriers to brain drug delivery, including the blood-brain barrier, intranasal administration emerges as a non-invasive alternative. Notably, intranasal insulin has shown neuroprotective effects, impacting Aβ clearance, tau phosphorylation, and synaptic plasticity. In preclinical studies and clinical trials, intranasally administered insulin achieved rapid and extensive distribution throughout the brain, with optimal formulations exhibiting minimal systemic circulation. The detailed mechanism of insulin transport through the nose-to-brain pathway is elucidated in the review, emphasizing the role of olfactory and trigeminal nerves. Despite promising prospects, challenges in delivering protein drugs from the nasal cavity to the brain remain, including enzymes, tight junctions, mucociliary clearance, and precise drug deposition, which hinder its translation to clinical settings. The review encompasses a discussion of the strategies to enhance the intranasal delivery of therapeutic proteins, such as tight junction modulators, cell-penetrating peptides, and nano-drug carrier systems. Moreover, successful translation of nose-to-brain drug delivery necessitates a holistic understanding of drug transport mechanisms, brain anatomy, and nasal formulation optimization. To date, no intranasal insulin formulation has received regulatory approval for AD treatment. Future research should address challenges related to drug absorption, nasal deposition, and the long-term effects of intranasal insulin. In this context, the evaluation of administration devices for nose-to-brain drug delivery becomes crucial in ensuring precise drug deposition patterns and enhancing bioavailability.

**Graphical Abstract:**

Drug transport mechanism through the nose-to-brain pathway using the olfactory and trigeminal nerves (major pathway) and from the bloodstream through BBB (minor pathway).

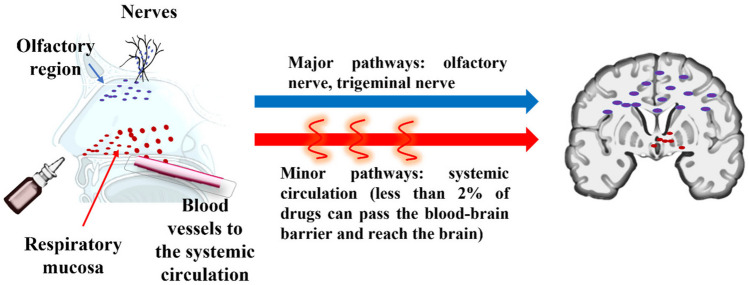

## Introduction

Alzheimer’s Disease (AD) is a central nervous system (CNS) condition that impacts many older adults. Approximately 44 million people worldwide are living with AD or other dementia [1], with AD representing 60–80% of all dementia cases [2, 3]. AD is characterized by alterations in amyloid beta (Aβ) and gradual degeneration of neurons, leading to a decline in cognitive function and disruption in daily routines [4]. However, the exact aetiology of AD remains unknown [2, 4, 5]. Over the past few years, a distinct correlation between diabetes mellitus (DM) and the risk of cognitive impairment has been demonstrated by several epidemiological studies [6, 7]. This link was found more present in adults with type 2 diabetes mellitus than in type 1 DM, in which glucose intolerance correlated well with AD in later life. People with T2DM have a heightened risk of 1.5-fold for cognitive decline acceleration and 1.6-fold for the occurrence of AD [2, 7]. The possible underlying mechanisms can be related to desensitization of insulin signalling in brains (i.e. attributed to obesity and T2DM), impaired insulin function in the CNS, and attenuated sensitivity of insulin receptors.

Specifically, insulin deficiency in the brain or cerebrospinal fluid and downregulation of sensitivity of insulin receptors in the brain have been reported in adults with AD [1, 6, 8]. A downregulation of insulin levels in the CNS can impair Aβ clearance and Aβ deposition, leading to increased neurotoxicity and impaired brain functioning [4, 8]. AD has now been referred to be a type of DM, known as type 3 DM, as insulin resistance is often also observed in the brain [9]. Pathologies in individuals with DM or cognitive impairment share common features. These include chronic exposure to inflammation markers and oxidative stress that is detrimental to brain health [7, 10]. It has been shown that impairment in either insulin or insulin-like growth factor (IGF)-1 signalling pathways can accelerate the progression of neurodegenerative disorders such as AD [11] and decreased permeation of peripheral insulin across the blood-brain barrier (BBB), leading to a shift in the proportion of brain to peripheral insulin levels [3, 6, 9].

Given the increase in the global prevalence of chronic medical conditions, including DM, obesity, and AD, a growing body of investigations has been focused on exploring the effects of intranasally administered drugs on the CNS to prevent and/or treat memory decay [6, 12]. Recent research has revealed that reduced insulin levels in the brain can negatively affect cognitive function [6, 11]. On the other hand, drugs with anti-diabetic properties, including insulin and glucagon-like peptide (GLP-1), have demonstrated efficacy in enhancing cognitive function in individuals with comorbidities (i.e. DM and AD). Insulin is a hormone typically secreted by the pancreatic beta cells that can mediate the metabolism of nutrients and glucose uptake at the molecular level. In contrast, GLPs are large hydrophilic incretins that can lower the risk of cardiovascular disease, body weight, suppress glucagon secretion and restore the production of insulin by the body [6, 7, 13, 14]. Importantly, both therapeutic peptides have demonstrated beneficial pharmacological effects on cognition and memory by reducing the chronic inflammatory response and re-sensitizing insulin signalling; these are generally administered by subcutaneous injections to exert therapeutic effects [7, 11]. Multiple pre-clinical studies have demonstrated the neuroprotective effects of insulin, insulin-like growth factor and GLP-1 analogues administered by the intranasal route in animal models of AD and PD, including a reduction in AD hallmarks (e.g. amyloid plaque load), restoration of synaptic plasticity, lowered chronic inflammation response, reduced functional impairment, and improved synaptogenesis or neuronal functionality [4, 7, 11, 15–17].

Drug delivery to the brain is critical due to physiological barriers, including BBB, enzymatic degradation, hepatic metabolism, and systemic elimination [4, 16]. BBB, a tightly composed endothelial barrier, comprises microvascular endothelial cells, astrocytes, and pericytes [18]. It is the primary barrier that restricts the influx of drug molecules to the brain from systemic blood circulation [9, 15]. Nearly all macromolecules fail to penetrate the BBB, lowering the therapeutic efficacy and amount of the drug reaching the brain [4]. Therefore, most of the available conventional treatments for AD utilize oral and parenteral routes, which present limited drug potency and bioavailability due to their inability to cross the BBB [4]. Hence, drugs with a fragile structure or low bioavailability in the CNS are required to be delivered directly *via* invasive and painful approaches, including intrathecal, intracerebroventricular and intraparenchymal injections [4, 15]. However, these invasive administration routes are accompanied by poor patient acceptability and the need for highly experienced health professionals for drug administration and have been associated with a well-documented risk of infections. In this context, intranasal administration, as a non-invasive route, is preferred over the systemic routes for drug delivery into the CNS, particularly for neurotrophic proteins, neuropeptide drugs and nucleic acids (e.g., mRNA, siRNA) [18]. Nasal delivery of the drug could circumvent the BBB and elicit a sufficient therapeutic response to treat neurological diseases, cerebral ischemic injuries, and brain tumours [6, 19].

This review examines the critical role and efficacy of intranasally administered insulin, elucidating its neuroprotective effects in individuals with both DM and pre-existing cognitive impairment. The transport mechanism of insulin from the nasal cavity to the CNS will be explored. Additionally, the review addresses the suitability of intranasal insulin and the challenges inherent in delivering protein drugs from the nasal cavity. Comprehensive coverage will be given to treatment strategies involving nasal formulations, including the utilization of formulation excipients such as tight junction modulators and cell-penetrating peptides. Furthermore, the discussion will extend to innovative nano-drug carrier systems, encompassing polymeric nanoparticles, solid lipid nanoparticles, nanogels, quantum dots, and phospholipid magnesomes, highlighting their role in re-sensitizing insulin pathways in the brain. The review will also illustrate nasal drug administration devices designed for nose-to-brain delivery of protein and peptide drugs. Lastly, future directions will be explored, drawing insights from pre-clinical and clinical trials, to illuminate potential avenues for enhancing drug efficacy targeting specific brain regions.

## Role and cognitive benefits of insulin in the brain

Insulin is primarily recognised for its peripheral effects such as regulating nutrient metabolism (glucose, fats, and proteins) and maintaining glucose homeostasis *via* cellular uptake and storage in skeletal and adipose tissue [6–8, 12, 20–24]. It also functions as a growth factor hormone that maintains autophagy, cell growth, energy utilization, mitochondrial function, oxidative stress management and protein synthesis [7, 25]. An elevated level of intranasally-administered insulin in the nasal cavity can reduce olfactotoxic drug-induced olfactory epithelium cell damage or p53-dependent apoptosis (e.g., methimazole, eosinophilic cationic protein) [8, 26, 27]. Yet, insulin also functions as a neuropeptide and exerts important CNS actions including controlling peripheral nerve function, neurogenesis, nerve activity, neuronal plasticity, calorie homeostasis, glucose regulation, energy balance, regulation of lipolysis in adipose tissue, synaptogenesis, synaptic remodelling, cognitive function, learning, human memory, food intake, reproduction, growth and endocrine function [1, 6, 8, 12, 28–30]. Central insulin level can affect glucose homeostasis and neuronal activity, in which a high concentration of insulin in the CNS serves as a positive feedback loop for insulin secretion in the periphery *via* vagal neuronal control and reduces food intake [6]. Therefore, a de-sensitization of insulin signalling in brains can compromise energy utilization and growth factor signalling [7].

## Suitability of intranasal insulin with antidiabetic property as a neuroprotein for neurological disorders

The brain is an organ that is both insulin- and glucose-sensitive [8]. Insulin receptors can be found predominantly in brain regions including olfactory bulbs, the hypothalamus (mostly in the arcuate nucleus), the cerebellum, the cortex, synaptic areas, and the amygdala. They are also located in the hippocampus and limbic system, which are the brain areas responsible for memory [4, 6, 8]. The localization of insulin receptors in the olfactory bulbs heightens its significance for nose-to-brain delivery of therapeutic proteins or peptides such as insulin [6]. A surge in the brain concentrations of insulin and IGF-1 can smoothen neurotransmission and enhance nerve growth, which helps maintain declarative memory [4, 28]. Compared to plasma insulin, the amount of insulin present in the brain may range from 10 to 100 times more concentrated [28, 29]. Specific insulin receptors in the BBB, involving the formation of receptor-insulin complex and cellular internalization, can also affect the transportation of insulin from systemic blood circulation to the brain [8].

Intranasal administration of neuroproteins and neuropeptides offers an alternate way to manage neurodegenerative diseases such as AD. Other administration routes for drug delivery to the CNS are impractical regarding consumer compliance, cost-effectiveness, and safety [5]. In the nasal cavity, several protein moieties, including insulin and IGF (7.65 kDa protein), have been found in the mucus, mucosa membranes, nasal polyps, and olfactory mucosa [28, 31]. Insulin can be secreted from the serous glands and form a major component of the mucus fluid [28], making the nasal cavity a suitable location for insulin administration [6, 31]. The presence of endogenous insulin in nasal mucus can influence the functioning of sensory neurons in the olfactory region through signalling pathways that involve the insulin receptors [26]. Moreover, the degree of endogenous insulin secretion can vary in individuals. For example, a lower level of insulin is present in the nasal mucus for individuals who are non-fasting, diabetic and obese status [28]. In addition, the elimination of insulin from the brain can be initiated by insulin-degrading enzymes in the CNS or drainage of cerebrospinal fluid to the lymphatic system or venous blood vessels [25].

Recent studies revealed that several insulin-related protein moieties with low molecular weight (e.g. basic fibroblast growth factor, H102 peptide, nerve growth factor, pentapeptide, V24P peptide) can enter the brain within minutes through the transneuronal and the extraneuronal pathways [4, 8, 28, 29]. When drug moieties are instilled into the nasal cavity, they can permeate across the epithelial cells located in the olfactory region of the nose and enter the brain through the cribriform plate, followed by the olfactory bulb, brain parenchyma and cranial nerves [4, 6, 28, 32]. Intranasally administered drugs can also cross the BBB by reacting with receptors, allowing more drugs to be transported to the CNS with fewer peripheral side effects. Protein or peptide drugs, such as insulin, melanocortin, nerve growth factor, oxytocin and wheat germ agglutinin, given as a nasal spray, can reach brain areas including the hypothalamus, supporting the concept of nose-to-brain drug delivery [4, 33]. Exogenously administered insulin is regarded as a neuroprotective agent, and its delivery to the brain can enhance declarative memory in individuals with AD by modulating the insulin level in the CNS [6]. The beneficial effects of nasally administered insulin in the CNS have prompted researchers to examine its effects in managing neurological disorders.

As impairment in the insulin signal transduction pathway has been pinpointed as a distinguished hallmark of neurological disorders, recent research has focused on formulation strategies to re-sensitize the signalling pathway [7]. The therapeutic potential of intranasally administered insulin *via* the nose-to-brain pathway is prominent for AD, as it reduces peripheral adverse events (e.g., interference with peripheral glucose homeostasis) by minimising the distribution of the drug in the systemic blood circulation [6, 7, 11, 12, 34]. Hence, the blood glucose level can be maintained in the euglycemic range [8, 11]. The use of insulin is pivotal in the management of neurological disorders other than diabetes [31]. The neuroprotective effects of nasally administered insulin that have already been noted in slowing down the progression of neurological conditions, such as AD, anxiety, depression, eating disorders, olfactory epithelium injury, Parkinson's disease, Phelan-McDermid syndrome and substance addition, are independent of peripheral insulin concentration [6, 26, 29, 30, 35]. A high level of insulin in the CNS can induce insulin secretion from peripheral tissues and reduce blood glucose in systemic circulation [8]. The amount of endogenous insulin that accumulates in the brain is independent of peripheral blood glucose levels [28]. In clinical trials, delivery of insulin *via* the nasal route has shown promising results that align with the multifaceted actions of insulin in the CNS, for instance, an improvement in learning, performance of tasks on declarative memory, and verbal information [6–8]. Its beneficial effects on cognitive function and memory recall in individuals with mild cognitive impairment are mostly attributed to enhanced energy metabolism in neurons, neurotransmitter release and prolonged potentiation [31]. Some recent studies revealed that intranasally administered insulin affects the elimination of the Aβ peptide and tau phosphorylation, which are fundamental aspects in the pathogenesis of AD.

Moreover, the universality of intranasal insulin administration is underscored by its adaptability to varying respiratory conditions in different populations. Notably, individuals with respiratory conditions may exhibit alterations in the upper respiratory system, impacting the nasal delivery of intranasal therapies. While changes in the upper respiratory system may occur, they do not consistently result in decreased drug absorption [36]. For example, the presence of nasal polyps has been associated with extended residence time of compounds, and inflammation from chronic sinusitis or allergic rhinitis may enhance nasal mucosa permeability to drugs [37, 38]. Clinical studies suggested that the presence of rhinitis generally does not adversely affect absorption and bioavailability of intranasally delivered drugs such as zolmitriptan, hydromorphone, fentanyl and buserelin, reinforceing the versatility and efficacy of intranasal drug delivery [39]. However, further studies are required to comprehensively understand the effects of these respiratory conditions on the CNS bioavailability of intranasally administered drugs.

## Mechanism of insulin transport through nose-to-brain pathway

**Fig.1:**
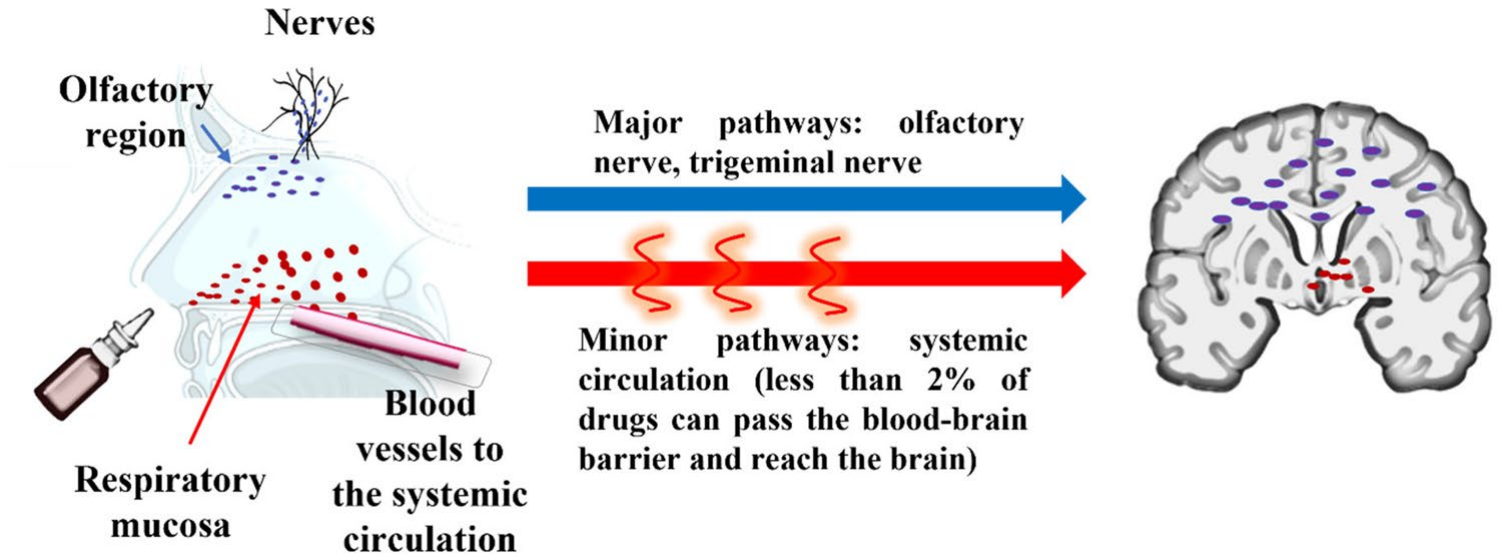
Drug transport mechanism through the nose-to-brain pathway using the olfactory and trigeminal nerves (major pathway) and from the bloodstream through BBB (minor pathway)

The intranasal administration route is an attractive needle-free option for treating many chronic conditions. Insulin can be transported to the brain *via* peripheral systemic circulation or administered directly into the brain [6, 8]. This is because the olfactory and trigeminal nerves are interconnected to the nose and brain compartments [3]. Several in vivo and human studies have supported the hypothesis that therapeutics (e.g. insulin, glial cell line-derived growth factor) can cross the nasal epithelial cells, and enter the brain through the olfactory system (i.e. nose-to-brain drug delivery), circumventing the BBB as they propagate the olfactory (major) or trigeminal routes (minor) [3, 5]. Figure 1 depicts the mechanism for nose-to-brain drug delivery. The drug must be deposited in the olfactory epithelium located on the apex of the nasal cavity for optimal delivery of proteins using this pathway for maximal transport *via* the olfactory receptor neurons [12, 13]. The intranasal route is particularly interesting for insulin due to the extensive insulin receptors-expression in the olfactory bulb [8]. Indeed, it has been known that intranasally administered insulin can promote positive CNS-related and behavioural effects in terms of weight management and memory [28]. Notably, upon intranasal administration, insulin can enter the systemic circulation and traverse both the blood-brain barrier (BBB) and the blood-cerebrospinal fluid barrier (BCSFB). These processes occur through a saturable transcellular transport mechanism facilitated by the presence of insulin receptors. This insight sheds light on the potential pathways through which insulin can reach the brain [40].

After insulin administration through the nasal route, multiple transport pathways can be involved in drug transport from the nasal cavity to the CNS. The efficacy of drug transport is determined by factors, such as the physicochemical properties of drugs and excipients (e.g., particle size, charge, solubility, lipophilicity, stability, solubility, absorbability and penetrability), viscosity in the formulation, the physiological environment, and the nasal administration devices [4, 5]. Based on the transport mechanisms proposed in previous literature, particles larger than 10 μm typically deposit fully in the nasal mucosa and become immobilised in the nasal mucus [41]. Also, the permeation of unionised molecules across the nasal mucosa membrane is more noticeable than ionised species as they tend to partition from aqueous into organic phases [41], eliciting biological effects within 30–80 min [3, 12, 13, 16]. Insulin and IGF-1 can also enter the brain through systemic circulation by specific receptors in BBB through a saturable carrier-mediated transport process (minor pathway) [4, 6, 35, 42, 43]. However, this pathway only has a minor contribution, with less than 2% of drugs entering the CNS from the bloodstream [9, 43].

## Challenges to deliver protein drugs from the nasal cavity to the brain

Delivering proteins specifically from the nose to the brain presents significant challenges due to several physiological barriers and mechanisms that impede efficient transport. Enzymes, tight junctions, mucociliary clearance (MCC), and precise drug deposition at the olfactory region are among the key factors contributing to these challenges [44]. Proteolytic enzymes such as peptidases present in the nasal mucosa can rapidly break down proteins, whereas tight junctions between nasal epithelial cells restrict the passage of large molecules and limit their entry into the brain. While it has been hypothesized that the mucociliary clearance in the olfactory region may be lower compared to other parts of the nose, drug navigation to this specific region remains a significant challenge due to the complex anatomy and its limited accessibility. The lipophilic nasal membrane poses an additional challenge by hindering the absorption of hydrophilic compounds like insulin, further underscoring the necessity for a suitable drug delivery system.

## Strategies to improve the therapeutic efficacy of intranasal drug delivery to the brain

### Formulation excipients

To optimise the bioavailability of nasal formulations, recent research focuses on addressing the fundamental limitations associated with intranasal drug delivery routes, such as mucociliary clearance and low drug permeation [4]. These include the incorporation of formulation excipients (e.g., tight junction modulators, cell-penetrating peptides) and the development of drug delivery systems (e.g., nanoparticles, liposomes) (Table 1) [4]. In terms of nano drug carriers, diverse formulations, including the self-emulsified nanoemulsion system [45], chitosan/alginate nanoparticles [46], phenylboronic acid-functionalized glycopolymer [47], chitosan-coated multivesicular liposome [48] and dipalmitoylphosphatidylcholine liposome [49], have been utilized to encapsulate insulin for intranasal administration. Notably, these studies have predominantly focused on investigating their potential hypoglycemic effects in diabetic animal models. This underscores the necessity for additional research to explore their efficacy in nose-to-brain drug delivery for Alzheimer's disease, specifically addressing the challenge of targeting the brain.

**Table 1:** Strategies to improve the CNS delivery of protein or peptide drugs

**Type of compound**	**Examples**	**Possible mechanisms of action**
**Tight junction modulator**	Clostridium perfringens enterotoxin mutant 194 [34, 50]	Disrupt tight junction barrier by binding the tight junction proteins
	Chitosan [13, 19, 41, 51–54]	Loosen the tight junctions transiently via redistribution of proteins such as cytoskeletal F-actin, occluding and ZO-1 proteins
	Saponins [55]	Formation of mixed micelles that promote drug absorption via the paracellular pathway
	Alkylglucosides, glycosylated sphingosines, oxidized lipids, and ether lipids [56]	Alter the lipid raft structure
	N-acetyl-L-cysteine [56]	Reduce the mucus viscosity
**Cell-penetrating peptide**	TAT, oligoarginines, low molecular weight protamine, HIV-1 Tat, penetratin, pVEC [57]	Facilitate the drug translocation into the cellular compartment by endocytosis
**Nanocarrier**	PLGA NPs [3], SLNs [3], chitosan NPs [3, 4], Poly (N-vinyl pyrrolidone) nanogel [16], phospholipid magnesome [58]	Enhance drug entry to the CNS through passive or active endocytosis mechanisms

#### Tight junction modulator

The paracellular pathway in the nasal mucosa is highly regulated by tight junctions, which function as a barrier against the free flow of foreign substances *via* distinct transduction pathways in the apical side of epithelial cells [50, 51]. Tight junction proteins including claudin-1, -4, -7, -8, -12, -13, -14, occludin, JAM-A, zonula occludens, and tricellulin, are involved in epithelial barrier function and regulation of the tight junction [34]. The tight junction proteins determine the permeation characteristics of the nasal epithelial layer and control the diffusion of ions and macromolecules across cells [41, 59]. Tight junction modulators can reversibly open tight junctions to enhance drug transport post-intranasal administration, [56]. These tight junction modulators can be classified into natural polymers, tight junction-modulating lipids, and tight junction-modulating peptides.

##### Natural polymers: chitosan

Among all the tight junction modulators, chitosan has been the most investigated natural cationic polymer for improving the nasal absorption of proteins for brain targeting [19, 56]. It is a linear polysaccharide composed of randomly distributed β-linked D-glucosamine and N-acetyl-D-glucosamine. Chitosan demonstrates biocompatibility, biodegradability, mucoadhesiveness, minimal toxicity, and permeation-enhancing properties. It shows affinity to the anionic components of the mucus membrane through electrostatic interaction, loosening the tight junctions transiently via redistribution of tight junction proteins such as cytoskeletal F-actin, occludin and ZO-1 proteins [41, 52, 53, 56]. In addition, chitosan can improve drug stability against enzymatic degradation, provide controlled drug release and offer a long contact time for protein absorption before removal of the nasal formulations by mucociliary clearance. The absence of chitosan toxicity in nasal epithelia has been confirmed in several animal studies (up to 28 days) as well as clinical trials (7 days), with no interference on nasal cilia beat frequency and bio-membranes [41, 52, 56, 60]. As such, chitosan has been adopted as a carrier matrix to prepare different nasal formulations, including hydrogels, thermosensitive *in-situ* gel, interpenetrating gel of glutaraldehyde-crosslinked chitosan, chitosan aqueous solution, chitosan powder, and chitosan microspheres [13, 19, 41, 51–54]. The discrepancy in the effectiveness of a copolymer in tight junction opening and bioavailability can be explained by the different polymer concentrations, molecular weight, chemical conjugation, degree of deacetylation, and the ratio of drug and polymer [32, 51, 61].

The pH of the nasal formulation does not only affect the electrostatic interaction between chitosan and therapeutic drugs but also their solubilities [32]. The suitable pH range for nasal drug administration is deemed to be between pH 4.5 and 7.4 [32]. The key limitation of using chitosan in a nasal formulation is that its poor solubility can lead to precipitation at pH 6 and above, losing the capacity to enhance drug permeability. To address these limitations, Gao et al. designed a drug delivery system using glyceryl monocaprylate (GMC)-modified chitosan as an enhancer to facilitate the absorption of insulin intranasally [32]. At optimal pHs, insulin can be encapsulated within the GMC polyelectrolyte complexes via electrostatic interaction, resulting in a decreased free-drug ratio [32]. As compared to unmodified chitosan, polymers (0.15% w/v) conjugated with GMC at a substitution degree above 12% demonstrated the strongest absorption-enhancing effect and more prolonged therapeutic effect for at least 5 h in vivo, which was attributed to improved cellular affinity to the nasal cells by the hydrophobically-modified group (GMC) [32].

Apart from chitosan, several other polymers, such as polyamidoamine, carbopol, PEG, poly(acrylic acid), poly-L-lysines, protamine, histone, polycationic dextrans, starch and saponins, have been explored for the administration of protein drugs *via* the nasal route [13, 41, 55]. In terms of saponins, Sajadi Tabassi et al. revealed that nasal formulations containing ATS (1% w/v) extracted from the root of Acanthophyllum squarrosum were more effective than QTS and SC in promoting insulin absorption through the nasal route [55]. Their capabilities in enhancing nasal absorption were ascribed to multiple mechanisms, including (i) the formation of mixed micelles at a concentration above its critical micelle concentration that enhances the transepithelial absorption of insulin, and (ii) the promotion of drug absorption *via* the paracellular pathway. Drug carriers lacking positive charge, like starch, can display shorter half-times of clearance in the human nasal cavity [53]. The improved efficacy in nasal drug absorption has been suggested to be related to the extraction of water from the nasal environment or alterations in the expression of tight junction proteins, forcing the opening of tight junctions in the paracellular pathway [52]. Although these polymers could prolong drug release and promote sustained therapeutic effects, constraints related to reduced drug biological activity and mucociliary clearance have been identified with the application of these polymers in nasal formulations [56].

##### Tight junction-modulating lipids and peptides

Tight junction-modulating lipids, including alkylglucosides, glycosylated sphingosines, oxidized lipids and ether lipids (e.g. coconut fatty acids), have been identified as tight junction modulators as they can alter the lipid raft structure [56]. However, some excipients displayed high cytotoxicity even at low concentrations (i.e., alkylglucosides 0.2–0.4%). Tight junction peptides can open the tight junctions reversibly and safely, reducing the transepithelial electrical resistance (TEER) across the nasal epithelium. This is beneficial for enabling the immediate onset of drug action and facilitating the paracellular transport of drug molecules with low molecular weight [56]. The tight junction opening effect in the nasal epithelium could be attributed to the activation of ZO-1 tyrosine phosphorylation. Several peptides with low cytotoxicity, including PN159, YY336, H-FCIGRL-OH and Zonula occludens toxin, have been evaluated *via* the intranasal route to enhance the absorption of therapeutic proteins in vivo [56].

##### Other tight junction modulators

Substances such as Clostridium perfringens enterotoxin (C-CPE) mutants, can attach to the tight junction proteins (e.g. claudin-4) and provoke disruption of the tight junction barrier as indicated by a significant drop in the TEER values in HNECs at 24 h [34, 50]. Once the C-terminal fragment of CPE binds to the receptors of tight junction proteins, it can induce an alteration in the permeability of the nasal membrane due to complex formation in a time-dependent manner [34]. The C-CPE mutants significantly improved the extent to which insulin permeated the nasal cells through ERK1/2 phosphorylation in the MAPK pathway [34, 50]. The disruption of the barrier did not induce any cytotoxic effects or alter the localisation pattern of claudin-1, -4, -7 and occludin, suggesting its therapeutic potential and use in intranasal insulin therapy for neurological disorders such as AD [34, 50]. However, its feasibility in forming a conjugated complex with a drug or as a drug carrier will require investigation. Last but not least, nitric oxide donors (e.g. S-nitroso-N-acetyl-DL-penicillamine, sodium nitroprusside) have been identified as important regulators of tight junction and can result in increased drug absorption across diverse epithelial cells [56]. However, their uses in enhancing the nasal absorption of protein or peptide drugs have not been fully evaluated.

#### Cell-penetrating peptide

The cell-penetrating peptides (CPPs), also often termed ‘protein transduction domains’, are a group of short functional peptide moieties (less than 20 amino acid residues) that can facilitate the intracellular transport of bioactive agents with poor permeation across the nasal epithelium in a dose-dependent manner [9, 13, 62–65]. CPPs are diverse in electric properties, size, hydrophilicity, primary sequences and secondary structure [57]. Thus, the cellular uptake efficiency and modes of promoting drug uptake can be different. The level of toxicity of CPPs is highly dependent on the peptide type, coupling process concentration, and cargo molecules [62]. Some examples include arginine-rich linear CPPs (e.g. TAT, oligoarginines), cationic CPP (e.g. low molecular weight protamine, HIV-1 Tat), and amphipathic CPPs (e.g. penetratin, pVEC) [57]. The universally accepted CPPs can facilitate the translocation of nasally administered drugs, such as DNA, small interfering RNA and proteins (e.g. GLP-1 agonist, leptin, insulin), into the cellular compartment by endocytosis without intervening with receptors [57].

Co-administration of CPPs in a physical mixture of the nasal formulation can facilitate nose-to-brain targeting of protein or peptide drugs utilizing transcellular pathways through the olfactory epithelium or axonal transport along the olfactory nerve [18, 43]. In the presence of L-penetratin, Kamei et al. confirmed that the olfactory mucosal pathway initiated nose-to-brain transport of insulin and Exendin-4 but not the trigeminal nerves [18]. Fifteen minutes after intranasal administration of L-penetratin formulations, autoradiography showed that the radioactive insulin was first distributed in the olfactory bulb, followed by the anterior part of the brain [43]. This was consistent with the results obtained by Kamei et al., in which the distribution of insulin first originated from the olfactory bulb, and subsequently found to accumulate within the hippocampal neuronal cells through the cerebrospinal fluid [18]. These findings confirmed that intranasally administered insulin in the presence of CPP reached the anterior section of the brain (e.g., brain stem, cerebellum and cerebral cortex) with the potential to target specific brain areas for memory improvement [9, 43]. The use of D-form CPPs to form drug complexes can be more resistant to enzymatic degradation, reducing the degradation rate of protein drugs by peptidases for better drug absorption [62]. However, the effects of some CPPs such as penetratin can vary in different isoforms, specifically a higher cell internalization efficiency in L-form for systemic drug absorption and better drug delivery efficacy to the olfactory bulb with less systemic exposure using the D-form [9, 62]. Special care is required to understand CPPs-conjugated drugs' capability to escape from the endosome using different isoforms since improper design can lead to low drug release from the basal membrane [57].

CPPs can be conjugated into bioactive drugs (*via* electrostatic or hydrophobic interaction) or drug carrier systems such as nanoparticles, micelles, and liposomes [9, 57, 62]. CPPs with inherited cationic charges can enhance the capacity of the carrier to bind to the nasal epithelium by interacting with the anionic membrane constituents [62]. In addition, nanoparticles modified with CPPs (e.g. TAT-conjugated PLGA NPs) can display a more efficient transport of encapsulated drugs, facilitating drug accumulation in the olfactory bulb and cerebrum [57]. Under optimised concentration and peptide-to-drug ratio, the CPPs have been demonstrated to improve the delivery of hydrophilic macromolecules, such as GLP-1 or insulin, to the brain without compromising the bioactivity of drugs or nasal cell integrity, both in vitro and in vivo [13, 43, 56, 62, 63]. When covalently conjugated to a carrier or active drug, CPPs such as Tat peptide (CGGGYGRKKRRQRRR), penetratin (amphipathic CPP) and octa-arginine (non-amphipathic CPP) can facilitate the translocation of drug intracellularly across the nasal epithelial cells through various endocytic pathways (e.g. clathrin- or caveolin-mediated endocytosis, macropinocytosis), improving the permeation of proteins by at least 6 to 8 times [9, 13, 62, 66]. However, concerns should be addressed regarding the reduced function of bioactive drugs, which can be associated with the conjugation of the drug structure with CPPs [65].

Suboptimaldevelopment of formulation with CPPs can lead to immunogenicity and subsequent safety issues [57]. Although improving the drug permeability across the nasal mucosa is often correlated to damage to the nasal cell membrane, formulations that were incorporated with CPPs have had negligible undesirable effects on the immunogenicity, the release of lactate dehydrogenase from nasal perfusate and histopathological integrity of nasal respiratory epithelium [57, 62, 63]. To date, controversies exist regarding the detailed mechanism of CPP transport from the nasal cavity to the CNS, cell binding affinity, brain distribution, and their biological effects (e.g., Aβ level, progression of neurodegeneration). A more comprehensive understanding of the transport mechanism is required to increase the drug bioavailability and safety profile of formulations containing CPPs [65]. Some cell-penetrating peptides, such as MIIFRALISHKK [64, 66, 67], are effective strategies for enhancing the systemic absorption of insulin in the nasal cavity, but their effects on the delivery of drugs to the brain and transport mechanisms are yet to be understood [65]. Further investigation on mitigating the delivery of biopharmaceuticals from the systemic circulation, while increasing the efficacy of brain delivery using CPPs *via* an axonal route along the olfactory (to reach the olfactory bulb) or trigeminal nerves (to reach the brain stem) will be required [9, 43].

### Nano-drug carrier system

Drug carrier serves a critical function in improving the absorption and delivery efficacy of protein drugs *via* the nose-to-brain pathway. The application of a suitable delivery system can improve the duration of drug retention and reduce the impact of mucociliary clearance [4]. To improve the CNS bioavailability of the drug, the nano-drug carrier system is one of the most widely adopted approaches, as it can enhance the cellular uptake of drugs, improve brain drug concentration, and lessen the systemic adverse events linked to the encapsulated parent drugs [4]. Only a handful of studies have reported using the nanoparticulate system in nasal delivery of fragile therapeutics [3]. Nanocarriers can facilitate drug uptake to the CNS through the nasal cavity's olfactory or respiratory pathways [3]. Nanoparticles with a size falling below 200 nm are ideal for drug uptake *via* the endocytic pathway to improve brain targeting [3, 4]. The particle size of the nanocarrier and its size distribution can be impacted by the polymer concentration, stirring rate, and pH of the solution mixture during the formulation process [4, 68–78]. Importantly, if encapsulated, a suitable nanoparticle system can reduce the enzymatic degradation (e.g., protease) of intranasally administered protein drugs.

#### Polymeric nanoparticles and solid lipid nanoparticles

Poly (lactic-co-glycolic acid) nanoparticles (PLGA NPs) and solid lipid nanoparticles (SLNs) hold great potential for the nose-to-brain delivery of protein or peptide drugs because of their abilities to enhance drug retention time, permeability, and overall CNS bioavailability. These nanocarriers have been documented to enhance drug entry to the CNS through passive or active endocytosis mechanisms both in vitro and in vivo [3]. However, their internalisation efficacy through the endocytosis pathways can be significantly limited in the absence of surface modification (e.g., chitosan-coating process, cell-penetrating peptides) [3]. Chitosan is a suitable candidate for designing a polymeric nano-drug carrier system *via* ionotropic gelation [4]. Chitosan nanoparticles offer favourable features including biodegradability, biocompatibility, mucoadhesiveness (i.e., for prolonged nasal retention time and reduced mucociliary clearance), better drug stability, controlled drug release (i.e., attributed to the hydration and swelling mechanism of polymer) and modulation of tight junctions. The use of chitosan nanoparticles for nasal administration can assure drug delivery to the brain [3, 4]. Compared to chitosan solutions, chitosan nanoparticles were proven to enhance insulin delivery to rabbits' CNS due to improved drug attachment to the nasal membrane [3, 79]. Along with nanotechnology, the efficacy of intranasal brain drug delivery can be facilitated by the incorporation of formulation modifiers, for instance, mucoadhesive polymers and cell-penetrating peptides [4]. Polymers with superior mucoadhesiveness can eliminate drug removal by mucociliary clearance, enabling an extended period for NPs absorption [3]. Csóka stated that chitosan coating over the insulin-loaded PLGA NPs or SLNs, with sizes of approximately 145 to 175 nm, promoted drug mucoadhesiveness and diffusion through the nasal mucosa [3]. Nanoparticles with a coating can improve the drug encapsulation efficiency, and better preserve the structural stability as well as biological activity of the encapsulated proteins. When compared to uncoated nanocarriers, both chitosan-coated SLNs and PLGA NPs presented more sustained drug release and higher mucoadhesiveness in the nasal cavity [3]. In addition, the surface-modified NPs promoted insulin permeation across both human nasal epithelial (RPMI 2650) and brain endothelial (hCMEC/D3) cells with no notable cytotoxicity, supporting their suitability for the intranasal application and nose-to-brain drug delivery.

#### Nanogel

Nanogels are submicron-sized hydrogels that consist of a three-dimensional network of crosslinked polymers capable of absorbing water or other solvents [80]. Poly (N-vinyl pyrrolidone)-based nanogels (NG) covalently conjugated with insulin (NG-In), with a particle size of approximately 70 ± 20 nm, can be synthesized by a simple and scalable procedure comprising e-beam irradiation [16]. PNVP is a mucoadhesive and biocompatible excipient with no known antigenicity [16]. The drug maintained its biological activity when insulin is embedded in the nanogel due to better resistance against proteolytic enzymes than free protein drugs [71]. Therefore, the delivery of drugs using nanogel to the hippocampus and cerebral cortex was more efficient [71]. Upon administering the insulin-conjugated nanogels *via* the nasal route, the formulations effectively enhanced the delivery of therapeutic proteins to the different brain regions in a mouse model [16]. No changes in immunological reaction (e.g., white blood cell and red blood cell counts) and morphological alterations in the liver and kidneys were noted [16].

#### Quantum dots

Quantum dots (QDs), semiconductor nanocrystals, have emerged as innovative carriers for their versatility in biological and bio-medical applications [81]. Recently, quantum dots have been introduced for brain delivery, marking a significant advancement in this field [82]. Their key attributes include tuneable size, versatile surface chemistry, and biocompatibility. QDs are fluorescent nano-carriers with narrow-band emission when excited by external lasers [83]. There has been a diversification of QD types, such as silver QDs, gold QDs, silicon QDs, and graphene QDs. However, most metallic QDs formed from compounds like cadmium and selenium raise concerns about toxicity [84]. On the other hand, graphene QDs have demonstrated capabilities to inhibit the aggregation of amyloid β peptides, which is a crucial factor in Alzheimer's disease [85]. Recent attention has shifted towards carbon QDs (CQDs) due to their non-toxic nature, solubility, cost-effectiveness synthesis, and eco-friendliness [81, 86]. Food-borne CQDs, which are derived from roast food items, while interacting with serum protein, present low toxicity, and high biocompatibility [87]. Their size, generally in the range of 100 nm, hold relevance for brain targeting and treatment of neurological disorders. In a study by Camlik et al. [81], the incorporation of a temperature-responsive mucoadhesive gel was explored for its effect on CQDs-loaded insulin. The resulting formulations, characterised by their small size (8–10 nm) and negative surface charge (-31.51 ± 1.59 mV), showcased high stability and minimal aggregation. The inclusion of Poloxamer 407 and methylcellulose in the *in-situ* gel formulation not only triggered gelation at body temperature, but also increased the residence time of insulin, facilitating complete drug release after 9 h in the nasal mucosa. Importantly, this formulation showed promising potential for bypassing the BBB, concentrating in nasal mucosa cells, thereby offering an alternative method for Alzheimer's disease therapy through nasal administration.

#### Phospholipid Magnesome

Natsheh et al. developed a novel nano-sized multilamellar nasal drug delivery system, called Phospholipid Magnesome, comprised of alginate (mucoadhesive polymer), magnesium ion, phospholipid, propylene glycol (fluidizing agent), and water [58]. This carrier system can load both lipophilic and hydrophilic molecules, hence is feasible for nose-to-brain delivery of CNS-acting drugs such as peptides, proteins, and small molecules. As verified by the in vitro mucoadhesiveness test using a nasal porcine mucosa membrane, the absorption efficacy of model proteins (epidermal growth factor, insulin, oxytocin) and small molecules (tramadol) was improved due to the enhanced mucosal contact time (i.e. mostly contributed to alginate) and absorption enhancement [58]. Phospholipid Magnesome presented a promising safety profile with negligible toxic effect (e.g., ulceration) on the integrity of nasal mucosal epithelium, and did not induce infiltration of inflammatory cells [58]. Thus, it unveiled a new approach to treating neurological diseases [58]. However, concern regarding formulation stability remains and can limit their practical application [4].

## Nasal drug administration devices for nose-to-brain drug delivery

Evaluation of the nasal delivery device is crucial in the formulation development stage as it can affect the formulation's deposition pattern in the different regions of the nose, drug clearance rate, and bioavailability [88]. The effectiveness of nasal drug delivery is determined by human factors (i.e. angle and force of actuation, nasal cavity structure) and spray characteristics (i.e. droplet size, plume angle, plume area) [13]. Drugs can be susceptible to mucociliary clearance if they are delivered to the nasal cavity [4]. When the volume of a spray applicator varies by 10%, it can create a difference in the therapeutic response and peak serum drug concentration [89]. As an example, an intranasal insulin spray (Nasulin™) was discontinued due to the disappointing and heterogeneous results in blood glucose management, which could be attributed to significant differences in the anatomical structure of the nasal cavity and the inconsistent drug deposition by nasal spray on epithelial cells [15].

**Fig.2:**
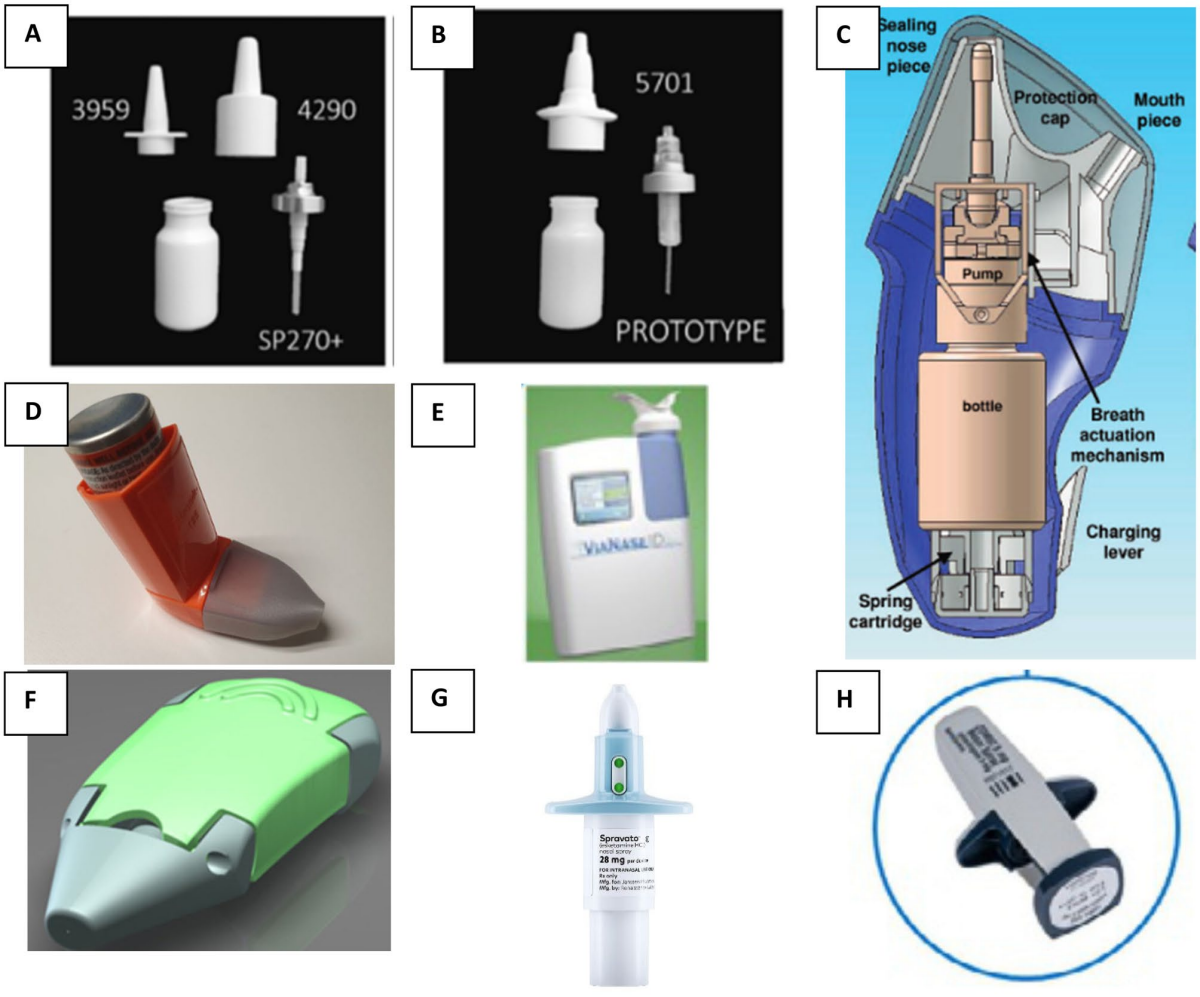
Nasal spray devices for administration of CNS therapies. **A** The SP270 + pump with 3959 or 4290 actuators [13]. **B** A prototype of the SP270 + pump with a prototype 5701 pump [13]. **C** A breath-actuated bidirectional delivery device (OptiMist™) [96]. **D** A metered dose inhaler with a printed nasal adaptor [97]. **E** ViaNase™ [98]. **F** Naltos [98]. **G** Spravato™ [98]. **H** Zomig [98]

Nose-to-brain delivery through the olfactory epithelium will require drug deposition to the upper part of the nasal cavity. Several nasal devices are available for administering liquid formulations (i.e. bi-dose nasal sprays, instillation catheters, metered-dose inhalers, nebulizers, squeeze bottles, unit-dose containers) and powder formulations (insufflators, mono- and multi-dose inhalers, pressurized metered-dose inhalers) [13, 90, 91], however, they do not necessarily deposit the drug to the olfactory region for brain targeting. The deposition site and drug clearance from the nose are heavily reliant on the type of nasal administration device [41]. A suitable nasal drug administration device (e.g., OptiMist™, ViaNase™) is critical for drug administration to the olfactory region in the nose [4, 92]. For example, ViaNase and Impel NeuroPharma I109 Precision Olfactory Delivery (POD^®^) devices have been developed for drug delivery to the olfactory cleft area for maximal CNS targeting [2, 92]. The use of a proper nasal administration device (~81.9%) can facilitate more drug retainment in the nasal cavity than the Pfeiffer device (~64.58%). Customised devices may be required for specific formulations such as viscous hydrogel or stimuli-sensitive formulations for effective administration into the nasal mucosa [93]. Gao and co-authors conducted a comprehensive review on the parameters influencing drug deposition in the nasal cavity [94]. In summary, they highlighted that the deposition of a nasal spray is contingent on various factors, encompassing device characteristics (such as spray angle, design, emitted dose volume, spray pattern, droplet size, and velocity of emitted droplets), formulation attributes (including viscosity, surface tension, and thixotropic properties), patient administration techniques (such as head orientation, administration angle, nozzle insertion depth, and breathing profile), and the physiological structure of the nasal cavity. Figure 2 illustrates the available devices used to target the drug to the brain. Further improvements in nasal drug delivery devices that deliver precise and reproducible drug doses could limit variation in the absorption rate in users [95].

## Animal study for nose-to-brain delivery of insulin

Alteration in insulin signalling in the brain has been proposed to be one of the contributing factors during AD progression [92]. Intranasal administration of insulin to the CNS can occur through the olfactory nerve pathway in animal models, modulating the level of insulin in the brain [99]. After nasal drug administration, behavioural changes and the overall health status of animals should be regularly and closely monitored [5]. The typical assessments to understand the effectiveness of CNS drugs employed in animal-based research include spatial memory (e.g., by water maze tasks) or instrumental learning (such as passive avoidance) [6]. Upon insulin administration to the CNS, multiple in vivo studies revealed that intranasally administered insulin was distributed throughout the brain quickly [1]. With the use of optimal formulation and proper intranasal administration techniques, Salameh et al. revealed that only 3% of ^125^I-insulin reached the systemic circulation of male CD-1 mice, hence no unwanted peripheral metabolic effects or changes in body weight were noted. Instead, ^125^I-insulin can target the brain *via* PKC inhibition and distribute in all brain regions, including the olfactory bulb (i.e., the highest concentration of uptake), hypothalamus, hippocampus and cerebellum within 2.5 to 60 min upon intranasal administration [1]. This finding is concordant with other studies showing that drugs can be distributed *via* the perivascular space surrounding the cerebral blood vessels [100].

Dimethylsulfoxide (DMSO), which is classified as a class 3 solvent with low risk to human health at the appropriate concentration, has been approved for use in pharmaceutical formulations [5]. It is widely employed as a carrier for drug delivery (transmitter) in AD-targeted therapy such as tau kinase due to its inhibitory and solubilizing effect on beta-amyloid peptides [5]. Maher et al. investigated the feasibility of a formulation combining insulin and DMSO for nose-to-brain targeting (patent number US8987199B2) in vivo. The developed formulation did not reach the lungs or the Sprague Dawley rats' systemic circulation but targeted all brain regions. Following a three-month successive treatment with insulin (2-3 µL per nostril; 4 IU and 6 IU), it was reported that insulin-DMSO significantly enhanced cognition in AD model senescence-accelerated mice. In addition, even at the high strength of insulin-DMSO formulation, no signs of drug-induced morphological lesions in nasal tissues or blood glucose level reduction were observed in the rats [5]. In the AD animal model, an altered insulin metabolism implicated the clearance of Aβ across the BBB. Intranasal insulin can prevent AD-like tau hyperphosphorylation in 3xTg-AD mice, a commonly used transgenic model of AD [1]. Insulin can reduce Aβ oligomer formation and protect against Aβ-induced synaptoxicity. Hence, it has been hypothesized that the administration of intranasal insulin to the brain can slow the progression of AD.

## Clinical trials for intranasal brain delivery of insulin

Research over the previous decade has indicated that the brain of humans is insulin-sensitive, in which dysregulation of insulin can contribute to the pathophysiology of AD [92, 101]. Data from both animal and human studies have established a clear link between peripheral insulin abnormalities (e.g., hyperinsulinemia, insulin resistance) and impaired neuronal function in AD [102]. Disturbances in brain glucose regulation, desensitization of insulin signalling, and reduced responsiveness to insulin or IGF serve critical roles in AD deterioration (brain insulin resistance) [92, 103, 104]. An impairment in the transport of insulin across the BBB can also contribute to a deficiency of insulin in the CNS. A downregulation of insulin sensitivity in the CNS, specifically the hypothalamus region, may further affect the release of insulin from pancreatic β cells and worsen insulin sensitivity peripherally in response to elevated levels of plasma glucose concentration [33, 105]. On the other hand, the supplementation of insulin and vitamin D levels in the CNS offered a synergistic effect in delaying the progression of cognitive decline and modulating AD pathology such as Aβ levels [106].

Based on the beneficial effects of insulin, randomised controlled trials (NCT01595646, NCT01767909) have been initiated to investigate the clinical efficacy of this therapeutic agent as a management option for AD [42]. Upon nasal application, insulin can promptly access the brain and exert its therapeutic effects [102]. An increase in insulin level in cerebrospinal fluid was noted within 10 min after administration of 40 IU insulin as nasal spray [107]. Most of the early studies used 160 IU of insulin to uncover the effects of nasal insulin delivery on blood glucose regulation, eating disorders and weight management, however, more recent studies have focused on the use of insulin with lower doses for memory and cognitive behaviour [107]. Importantly, nasally administered insulin at appropriate concentration can reduce the risk of side effects peripherally [101]. Multiple clinical studies have demonstrated consistent results and a clear relationship between intranasal insulin treatment and benefits in brain functions (memory, attention, reproduction of word lists) for healthy individuals (normal memory), those with the amnestic mild cognitive disorder (MCI) or AD, and older adults with memory impairment [1, 3, 16, 29, 92]. These trials mostly used regular insulin with more rapid action and a shorter half-life.

Dyslipidaemia is an important feature of insulin resistance that can alter β cell function and increase the risk of AD [108]. In a randomised controlled study, Xiao et al. examined the effects of intranasal insulin (lispro; 40 IU) on the synthesis of triglyceride-rich lipoproteins [108]. It was suggested that intranasal insulin that reached the brain played a significant role in the suppression of hepatic glucose production, although it had no effect on the secretion of triglyceride-rich lipoprotein from the liver or intestine [108]. These findings indicated that intranasal insulin at an administered dose adequate for hepatic glucose regulation was ineffective in controlling triglyceride-rich lipoprotein synthesis. Instead, a higher amount of insulin (160 IU) is required to reduce the level of circulating fatty acids. Rosenbloom et al. demonstrated that nasal insulin administration modulated brain activity with the strongest effect being noted after 160 IU [2]. However, such high concentrations of insulin can lead to pronounced spillover to systemic circulation and an increase in circulating insulin, inducing adverse side effects in peripheral tissues [2, 107]. More research will be essential to determine the possible effects of nasally administered insulin on complete lipid profiles such as low-density lipoprotein and plasma triglyceride [108].

However, patient response to the treatment can vary based on sex and ApoE ε4 allele for individuals with AD and MCI. For example, only men, but not women, showed significant cognitive improvement after the nasal administration of 40 IU insulin [102]. Treatment outcome for men with ApoE ε4 negative carriage was better with high doses of insulin, whereas insulin treatment negatively affected female participants with ApoE ε4 negative carriage [102]. Additionally, men who were administered intranasal insulin had more weight loss and reduced food consumption, while women gained weight due to a rise in extracellular water content and more sensitive negative feedback signal in adiposity control. These findings suggested that intranasal treatment may benefit populations with certain demographic backgrounds more than others. The variation in therapeutic outcomes could also be attributed to the difference in anatomy (e.g., distance from the nasal cavity to the brain), ethnicity, brain size and metabolic differences [102, 109]. Hence, a comprehensive understanding of the patient’s features, such as clinical background, can assist in the establishment of personalised therapeutic regimens and more accurate prediction of treatment outcomes.

Multiple types of insulin are available on the market that have diverse pharmacokinetic properties. Claxton et al. examined the effect of intranasally administered long-acting insulin analogues (insulin detemir) using a ViaNase delivery device on cognitive and daily function for adults with AD or MCI [92]. As insulin detemir with a long half-life demonstrates distinct pharmacodynamic profiles and sensitivity to insulin receptors, its therapeutic effect can differ from regular or rapid-acting insulin (e.g., glulisine) [103]. In vivo studies have demonstrated that BBB is not permeable to detemir due to its self-association properties (i.e. larger particles), and thus administration of insulin detemir *via* the intranasal route is required to ensure drug transport to the CNS [92]. A study demonstrated that patients, who are ApoE ε4 negative and overweight, are at risk of declined cognitive function due to insulin resistance [110]. Intranasal insulin detemir exerted better action in the hypothalamus and reduced peripheral insulin resistance in individuals who are APOE ε4 negative or obese, as compared to regular insulin. Over the 3-week course, it was reported that treatment with intranasal insulin (40 IU; 2 doses daily) significantly enhanced verbal and visuospatial working memory but not daily functioning or executive functioning in individuals with AD or MCI [92, 103]. However, in another randomised trial, Callens et al. reported that a longer duration of treatment (4 months) of insulin detemir (40 IU) had no significant effects on the cognitive functions of individuals diagnosed with mild to moderate AD or MCI, highlighting the risk for application of long-acting insulin in desensitizing the insulin receptors [110]. Nevertheless, caution is required when interpreting the findings from this clinical trial due to its small sample size. Further investigation will be required to delineate the long-term efficacy, mechanistic basis, responder characteristics and safety of insulin detemir with a larger sample size, particularly for individuals with memory impairment and APOE ε4 negative [92, 110].

Multiple studies have described the effective disposition of regular insulin and rapid-acting insulin (e.g., insulin aspart) in the CNS [109]. Lowe et al. performed an analysis on the central localization of intranasally administered insulin lispro in the cerebrospinal fluid of healthy participants [109]. In the study, two daily doses of insulin (i.e., 48 or 80 IU in the morning and 160 IU in the afternoon) were administered using an Aero Pump. It was reported that insulin lispro successfully reached the blood circulation 30–120 min after dosing, but not detectable in cerebrospinal fluid [109]. The reason for the discrepancy in the effects of insulin lispro and regular insulin could be attributed to the small sample size, short treatment period, and demographic characteristics amongst different trials. Therefore, further examination is crucial to examine the significance of intranasal insulin in a clinical context. Similarly, in a randomised controlled trial (NCT02503501), Rosenbloom et al. administered 20 IU of insulin glulisine twice daily to 35 memory-impaired individuals (aged 50–90 years) for 32 weeks using an Impel NeuroPharma I109 Precision Olfactory Delivery (POD^®^) device [2]. The device is designed to facilitate the transport of CNS drugs *via* the olfactory and trigeminal neural pathways. However, the study reported that intranasal glulisine had no effects on cognition and mood. The authors proposed that as most participants (2/3) were ApoE4-positive carriers (i.e. higher risk of age-related cognitive decline), reducing the efficacy of intranasal insulin in terms of memory enhancement due to the downregulation of cytochrome oxidase activity for neurogenesis [2].

Evidence has demonstrated a link between insulin resistance and the biomarkers of AD such as β-amyloid peptide [3, 111]. It is postulated that insulin possesses a multifunctional role in CNS, which involves the removal of Aβ peptide, tau phosphorylation, and restoration of insulin action in the brain [3]. A reduction in CNS insulin level is associated with the progression of AD as insoluble Aβ accumulates in the brain’s parenchyma and vasculature and induces synaptotoxic effects [111]. The restoration of CNS-related functions with intranasal insulin was believed to be related to protection against the adverse consequences of Aβ oligomers, reduction in Aβ accumulation and tau phosphorylation, and modulation of the tau protein-to-Aβ42 ratio in cerebrospinal fluid [42, 111]. In a randomised controlled trial, Craft et al. examined the influence of administering insulin (20 or 40 IU) through the ViaNase nasal delivery device on cognitive function and glucose homeostasis in individuals with AD or MCI over 4 months [111]. The beneficial outcomes of intranasal insulin in memory were proved to be attributed to improvement in the tau protein-to-Aβ42 ratio in cerebrospinal fluid, supporting future research to assess its clinical effectiveness for individuals with cognitive impairment over a longer period [111].

Overall, the clinical research performed thus far has indeed yielded promising results for intranasal administration of insulin, particularly for rapid-acting and regular insulin, in terms of the improvements in cognitive function [112]. In these trials, insulin was administered to AD or MCI patients with therapeutic benefits including word recall, delayed memory, memory recall and cognitive abilities. Recent trials have demonstrated the positive effects of nasal insulin in individuals with schizophrenia, bipolar disorder, depression, smoking addiction, and improving olfaction in individuals with impairment in the sense of smell [112]. Taken together, no treatment-related severe side effects have been noted, and most reported side effects were minor (e.g. mild rhinitis, nosebleed, dizziness, irritation, respiratory symptoms, blocked nose, hot sensation in nose, dry throat, headache, vomiting) [2, 92, 102, 109–112]. Nevertheless, additional studies are required to examine the duration of pharmacological effects as it is critical to maximise the therapeutic options in individuals with AD. Several studies identified that gender, age and ApoE genotype differences can lead to differences in treatment responses to intranasal insulin. Therefore, the experimental design in clinical trials (e.g., dosing, timing of the intervention) must be well-calibrated and follow the pharmaceutic industry standard, to ensure the administered drug does not spill over into systemic circulation.

## Future Direction

The successful translation of nose-to-brain drug delivery requires a comprehensive understanding of the drug transport mechanism, brain anatomy, and optimisation of nasal formulations. Although multiple studies favoured the use of intranasal insulin in humans after showing better cognitive function with few adverse effects, no formulation has gained regulatory approval for AD treatment due to a lack of clinical success and safety issues [4, 9, 56]. The nasal formulations must overcome the two major barriers that hamper insulin absorption across nasal mucosa, including low permeability due to large molecular weight and mucociliary action. Recently, researchers have mostly focused on the development of novel nasal formulations using excipients with minimal toxicity (e.g., absorption enhancers), but most investigations remain in the pre-clinical stages or early human clinical trials, which will require approval by the FDA. The long-term effects on adipose tissues at the application sites must also be considered. Studies have shown that decreased fatty tissue thickness at these sites, influenced by factors like temperature and insulin concentration, may lead to enhanced insulin absorption [113]. Addressing these potential negative effects is crucial for the development of safe and effective intranasal insulin formulations for the treatment of conditions like Alzheimer's disease.

The transport mechanism that drives the nasally instilled proteins, including insulin, IGFs and GLPs, into the brain is yet to be understood. Further studies are warranted to confirm whether nose-to-brain delivery of different protein or peptide drugs is contributed by direct drug targeting to the brain, indirect access through the BBB, or both. This will be crucial for selecting and optimizing nasal administration devices that can target the region of interest in the nasal cavity for optimal therapeutic effects [4].

The exact mechanism for insulin-induced memory improvement in different brain regions remains unclear [6]. For instance, the effect of intranasal insulin on tau proteins remains uncertain [3]. To unravel underlying mechanisms in vivo, experiments are essential to assess the influence of exogenous insulin on insulin signalling in the CNS. Further research to examine the long-term effect of nasal protein delivery on cognitive function in diabetic and non-diabetic adults is awaited [114]. As in vivo studies have demonstrated the neuroprotective actions of insulin in ameliorating 6-OHDA-induced motor behavioural impairments, the therapeutic potential of nasally administered insulin should be further explored in different neurological disorders such as PD and neuronal injury [100]. The long-term safety and toxicity of many novel polymers, such as CSGMC, on the nasal mucosa have not been examined, which will require further assessment [32]. The future research focus should be paid particularly to local nasal effect (e.g. irritation, congestion and rhinorrhoea), systemic effect (e.g. blood pressure, heart rate), and inflammatory response (e.g. insulin antibodies, T-cell proliferation) [13, 115, 116]. These adverse events can affect the patient’s compliance with the nasal formulations. Meanwhile, the drug clearance mechanism in the brain must be studied before the nasal product reaches the market [4].

To date, minimal details are provided to evaluate the parameters determining the effective dose for nose-to-brain drug delivery. These include the effective dose of insulin for re-sensitization of insulin signalling, choice of nasal device, spray angles, spray volume and deposition pattern [7, 13]. Although studies have demonstrated the potential of protein delivery from the nasal cavity to the brain parenchyma, challenges are still present to ascertain the precise quantity of drug solution required for intranasal administration for effective absorption in the cerebrospinal fluid or distribution in the brain tissues [35]. The use of physiologically relevant nasal casts and validated computational modelling will offer a more insightful understanding of the performance of nasal formulations, which is essential for optimal drug deposition in the right region of the nasal cavity and nose-to-brain drug transport [13].

Limited research has demonstrated the difference in the absorption efficacy between powder and solution formulations through the nose-to-brain pathway [117–122]. A study that directly compares the effect of different insulin forms (e.g. lispro, aspart, glulisine, regular, NPH, detemir) on brain targeting efficacy will be crucial, as recent studies revealed that fast-acting and regular insulin had better disposition in the brain than long-acting insulin [123]. The different forms of insulin can present different pharmacokinetics when applied nasally to individuals with different demographic backgrounds and medical conditions (e.g., colds and allergic rhinitis). Therefore, examining the targeting efficacy of drugs after nasal instillation and clarifying the impact of different drug forms and excipients on nasal absorption efficacy is important.

Several alternate peptides, including exendin, oxytocin, and pituitary adenylate cyclase-activating peptide, have presented enhancement in cognitive function after intranasal administration [1]. The use of alternate neuropeptide GLP-1 that can re-sensitize the insulin pathway in the brain could be an effective AD treatment [9]. GLP-1 has been a successful strategy to normalise insulin signalling in T2DM, which can stimulate the downstream of insulin signal transduction [9, 11]. Importantly, GLP-1 analogues have no impact on glycemia or desensitization of insulin receptors in people with normal blood glucose levels. Therefore, intranasal delivery of GLP-1 possesses great potential in treating neurodegenerative disorders in non-diabetic individuals, reducing Aβ plaque load and chronic inflammation in the CNS [7, 11]. The co-administration of CPPs can further facilitate the reversal of mild cognitive dysfunction by inducing neurite outgrowth and decreasing impairment in synaptic transmission [7, 11].

## Conclusion

The nose-to-brain pathway can effectively treat CNS-related disorders by increasing brain drug concentrations and bypassing the BBB while reducing interference with the systemic circulation. Innovative treatment strategies are leveraging nasal formulations to deliver drugs to the brain for individuals with cognitive impairment, with research focusing on nanocarrier-assisted delivery such as Lyospheres. The intranasal route possesses a relatively low burden that can increase patient compliance. Current findings are inconclusive in terms of the effectiveness of intranasal insulin as the sole treatment agent for individuals with AD or MCI. This was attributed to variations in therapeutic responses amongst individual patients and low drug bioavailability due to enzymatic degradation and rapid mucociliary clearance. Clinical trials with longer treatment durations and larger sample sizes will shed more light on the actual clinical significance of intranasally administered insulin on the cognitive functions of individuals with AD. Nevertheless, evidence is available to confirm insulin's bioavailability and safety profile as systemic adverse events such as hypoglycaemia are non-existent following nasal administration. Nose-to-brain delivery of peptide formulations requires proper formulation design to maximise the effectiveness of CNS drugs. Further clinical trials will be required to understand the long-term efficacy and side effects of intranasal insulin and its exact uptake mechanisms to the brain parenchyma. If findings from the clinical trials support the preclinical data, the intranasal formulation can be a potential breakthrough treatment option for AD.

## Data Availability

This is not applicable for a literature review.

## Acronyms

Alzheimer’s Disease: AD
Amyloid beta: Aβ
Blood-brain barrier: BBB
Cell-penetrating peptides: CPPs
Central nervous system: CNS
Diabetes mellitus: DM
Dimethylsulfoxide: DMSO
Glucagon-like peptide: GLP-1
Glyceryl monocaprylate: GMC
Mild cognitive disorder: MCI
N-acetyl-L-cysteine: NAC
Nanogels: NG
Nanoparticles: NPs
Poly (lactic-co-glycolic acid) nanoparticles: PLGA
Precision Olfactory Delivery: POD
Solid lipid nanoparticles: SLNs
Transepithelial electrical resistance: TEER
Type 1 diabetes mellitus: T1DM
Type 2 diabetes mellitus: T2DM
Type 3 diabetes mellitus: T3DM
